# Polystyrene microplastic contamination versus microplankton abundances in two lagoons of the Florida Keys

**DOI:** 10.1038/s41598-021-85388-y

**Published:** 2021-03-16

**Authors:** Susan Badylak, Edward Phlips, Christopher Batich, Miranda Jackson, Anna Wachnicka

**Affiliations:** 1 Fisheries and Aquatic Sciences Program, S.F.R.C., University of Florida, 7922 NW 71st, Gainesville, FL 32653 USA; 2 Materials Science and Engineering, University of Florida, PO Box 116400, Gainesville, FL 32611 USA; 3 South Florida Water Management District, 3301 Gun Club Road, West Palm Beach, FL 33406 USA

**Keywords:** Environmental impact, Structural materials

## Abstract

A microscopic study of microplankton in two coastal lagoons in the Florida Keys coincidently, and unexpectedly, revealed the widespread presence of high concentrations of polystyrene microplastic particles. The polystyrene particles were first observed in the second year of a 2-year study of phytoplankton communities, with peak densities in the spring/summer of 2019 at all ten sampling sites in the two lagoons. Polystyrene particle densities reached levels up to 76,000 L^−1^. The particles ranged in size from 33 to 190 µm, similar to the size range of microplanktonic algae (20–200 µm). Over the period of peak polystyrene densities, average particle densities were similar to average densities of microplanktonic algae cells. The latter observation highlights the potential significance of the microplastic particles for the ecology of the pristine waters of the Florida Keys, if they persist.

## Introduction

Since the development of synthetic polymer production in the early twentieth century the disposal of plastic materials into the environment has grown exponentially throughout the globe^1–3^. Plastic materials are carbon-based polymers made mostly from petroleum. Unfortunately, plastics have very slow rates of degradation, which can result in persistent presence in the environment for decades to millennia^2,4,5^. The United Nations Environment Programme considers plastic contamination a critically important environmental issue facing the global community^6^. Regionally, population size and waste management practices influence the output of plastic debris into the marine environment and without infrastructure improvements the quantity released into the oceans is expected to continue its increase^7^.

Plastic waste in the environment comes in many visibly obvious forms, known as macroplastics, such as plastic bags, drinking straws, milk jugs, rope, netting, food utensils and containers, floats, plastic bottles and cigarette filters^8–11^. The scope of macroplastic accumulation is evidenced by littered shorelines around the world. While macroplastics are the most visible components of plastic contamination very small particles, known as microplastics, may be just as important^3,12^. One of the initial observations of microplastics was in the early 1970s when small plastic particles were observed in water samples collected with a plankton net^13^. At the first international microplastic workshop plastic particles less than 5 mm in diameter were defined as microplastics^14^. In 2012, Hidalgo-Ruz et al.^15^ suggested two size classes of microplastics, 1µ–500 µm and 500 µm–5 mm, for the purpose of reporting. Since their discovery it has become obvious that microscopic plastic particles are widespread in the ocean and have accumulated in pelagic and sedimentary habitats^5^.

Here, we report on an observation of high concentrations of polystyrene microplastics in two coastal lagoons of the Florida Keys, widely viewed as among the most pristine marine waters along the eastern seaboard of the United States. The observation coincided with a study of the impact of coastal freshwater discharges on the ecology of phytoplankton communities in the two lagoons. The size of the polystyrene microplastic particles observed in the water samples fell within the range associated with microplanktonic algae, 20µ–200 µm^16^. The peak numerical abundances of the polystyrene microplastic particles were in the same abundance range as microplanktonic algae. While not visually obvious, the presence of such high concentrations of microplastics, if persistent, may have environmental consequences, such as disruption of food webs^3,17–19^ and alteration of microbial processes^20^.

## Methods

### Site description

Two coastal lagoons in the Florida Keys were included in this study, northeast Florida Bay, and Barnes Sound (including Manatee Bay) in the southern-most region of the broader Biscayne Bay ecosystem. Both regions are shallow, with depths less than 2–3 m and are characterized by extensive seagrass beds and fringing mangrove communities^21,22^. The study region is located in the sub-tropical environment of south Florida, which is characterized by modest seasonal temperature differences, and a dry season (November to May) and wet season (June–October), which coincides with the tropical storm season. The lagoons are part of a biodiverse marine ecosystem which provides food, shelter and nursery grounds for numerous fish and invertebrate species, including sponges, shrimp, spiny lobster, stone crab, as well as wading bird and marine mammal populations^23,24^.

Northeast Florida Bay is directly influenced by watershed discharges from the Florida Everglades wetlands and experiences water exchange with the southeast regions of the bay, which is bordered by the upper Florida Keys. Barnes Sound is connected to Biscayne Bay via the Intercoastal Waterway, a navigational feature that extends over the east coast of Florida. Barnes Sound (including Manatee Bay) receives freshwater inflows from the C111 Canal that drains freshwater from Everglades coastal wetlands, which are adjacent to agricultural, industrial and urban lands of southern Miami Dade County.

**Figure 1:**
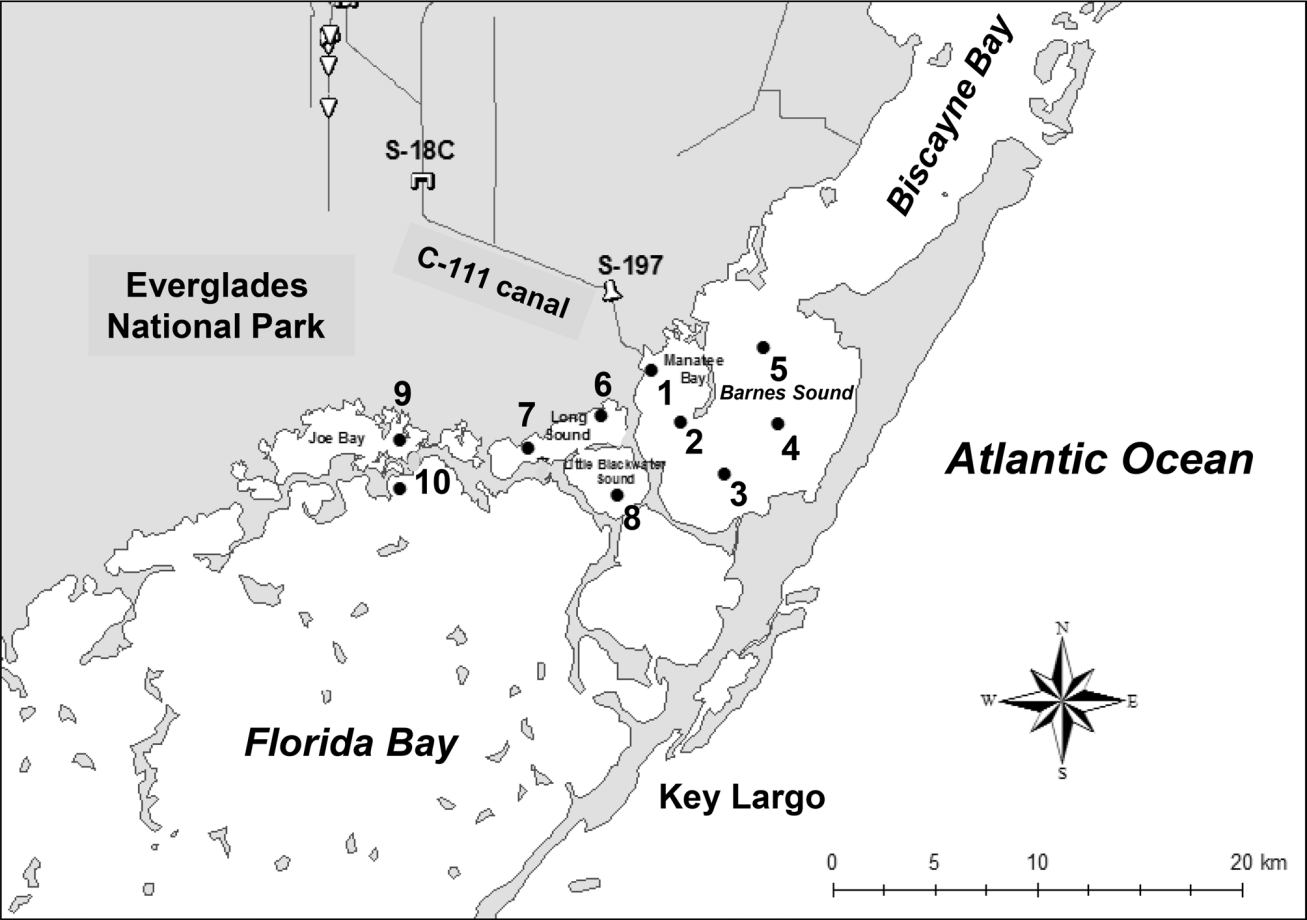
Map of sample regions and study sites (map was based on ArcMap Version 10.7 base image).

The sampling regime was originally designed to investigate the effects of water discharges from the land on downstream water quality and phytoplankton biomass and composition. Ten sampling sites were selected, five in northeast Florida Bay and five in Barnes Sound (including Manatee Bay) (Fig. 1).

### Sample collection

Twenty sampling events were carried out from May 2018 to April 2020 at ten sampling sites. Water samples were collected with an integrating sample pole (PVC) that captured water from the surface to within 0.3 m of the bottom^25^. Three pole samples at each site were mixed in a mixing vessel (HDPE) and aliquots were withdrawn and preserved with Lugol’s solution. The aliquots were kept in 125 mL amber glass bottles until microscopic analysis.

### Microscopic analysis

**Figure 2:**
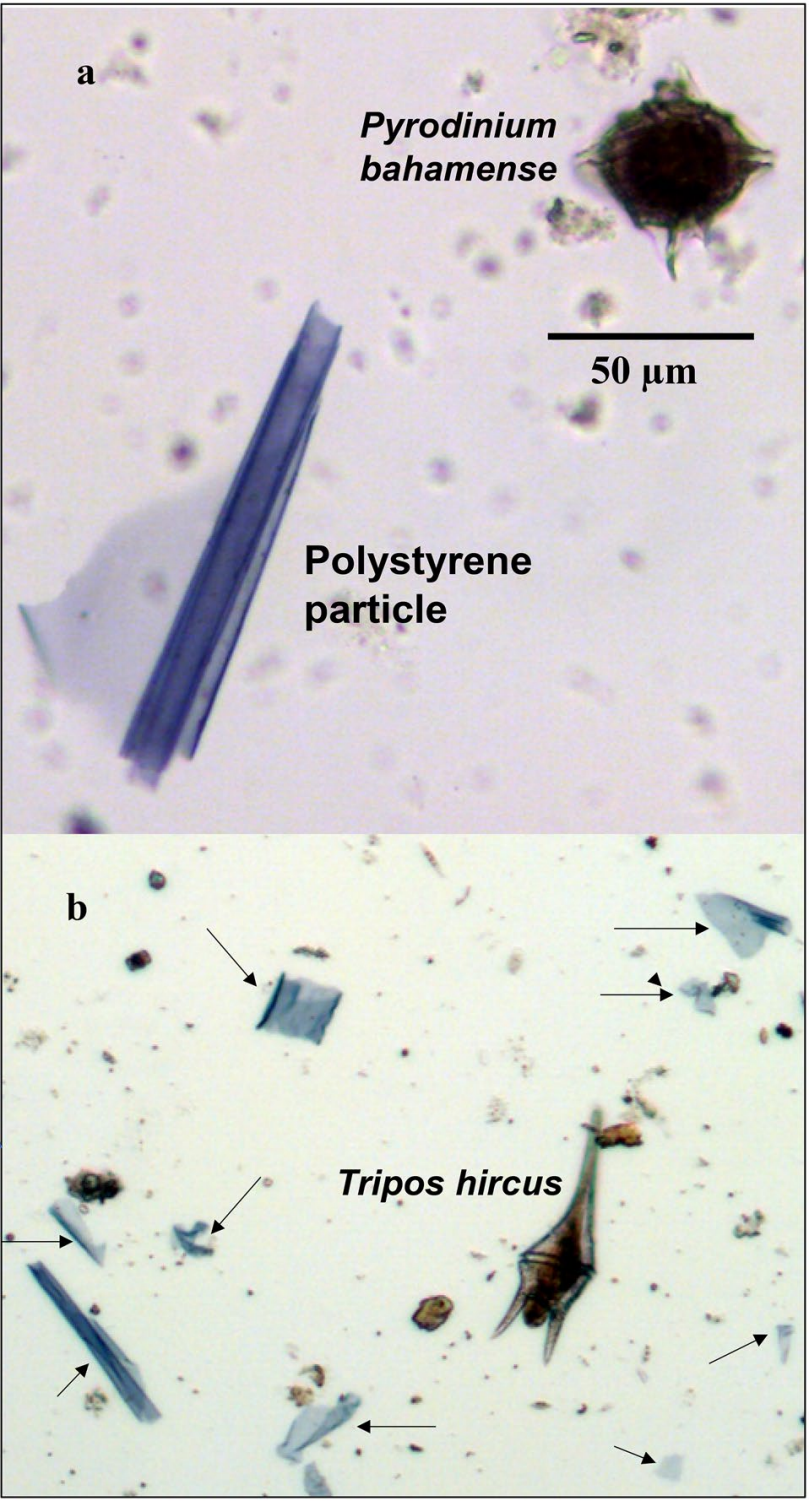
Microscopic image (×400 magnification) of a *Pyrodinium bahamense* cell and polystyrene particle in water sample (**a**—top panel), and microscopic image (×100 magnification) of a broader view of multiple polystyrene particles including the dinoflagellate *Tripos hircus*. Arrows indicate the location of polystyrene particles (**b**—bottom panel).

Prior to microscopic analysis, aliquots of the Lugol’s preserved samples were concentrated by pouring 100 mL of sample water into a graduated cylinder (HDPE) and allowed to settle for a week. Concentrated samples were drawn down to 20 mL, yielding a five-fold increase in concentration. The concentrated bottom 20 mL were kept in glass scintillation vials for plankton and plastic particle enumeration. To ensure all plastic particles had settled to the bottom of the graduated cylinder the 80 mL supernatant of a sample was examined for the presence of microplastic particles and no particles were observed microscopically. Microplanktonic algae (i.e. phytoplankton 20–200 µm) cells and microplastic particles were identified and counted in samples from at all ten sites and all twenty sampling events (May 2018 thru April 2020) with a phase contrast Leica inverted microscope using the Utermöhl method, a sedimentation procedure using a glass settling chamber^26,27^. 10 mL aliquots of the concentrated Lugol’s preserved samples were settled for a minimum of 24 h in a 19 mm inner diameter cylindrical glass chamber. Microplanktonic algae cells and microplastic particles less than 30 µm were counted at 400× magnification. Microplanktonic algae cells and microplastic particles greater than or equal to 30 µm were counted at 100× magnification. Microplanktonic algae cells and microplastic particles less than 30 µm were counted at 400× magnification. Only polystyrene microplastic particles were identified in the samples, as confirmed using Fourier-Transform Infra-Red microscopy (FTIR) analysis (see below). The polystyrene particles were easily identified as they all had similar structural morphology, color and thickness (Fig. 2).

For compositional analysis of the microplastic particles, two aliquots from the May 15, 2019 sampling event were analyzed. The microplastic particles in the samples were characterized with FTIR in reflectance mode. FTIR scans were performed using a Nicolet Continuum FTIR microscope employing an MCT-A detector, attached to either an iS50 FTIR optical bench or a Magna 760 optical bench. Spot size was approximately 100 × 100 microns except when limited for more linear samples. Samples were collected on Anodisc filters (0.2 µm pore size) and then brushed off that filter using a fresh camel hair bush onto a metal coated silicon wafer. This was used for reflectivity and flatness properties and was then placed on the stage of the microscope. The spectra obtained were baseline corrected and compared to authentic reference spectra in the spectral library. The particles showed a good match to polystyrene and not to other common polymers, such as polyethylene.

## Results

**Figure 3:**
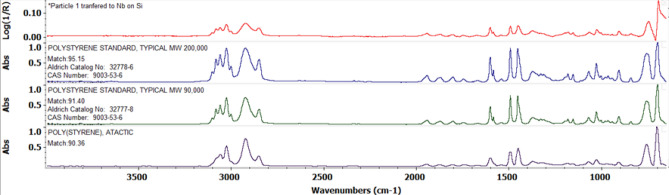
Baseline-corrected FT-IR spectrum of microplastic particle in water samples (top panel) particle and comparison reference spectra for standard forms of polystyrenes (the most common commercial forms). The percentage match for the three reference forms were: (1) 95.15% for Polystyrene Standard, typical MW 200.000 (HR Aldrich FT-IR Collection 1, (2) 91.40% for Polystyrene Standard, typical MW 50.000 (HR Aldrich FT-IR Collection 1, and (3) 90.36% for Polystyrene Atactic (Hummel Polymer Sample Library).

**Figure 4:**
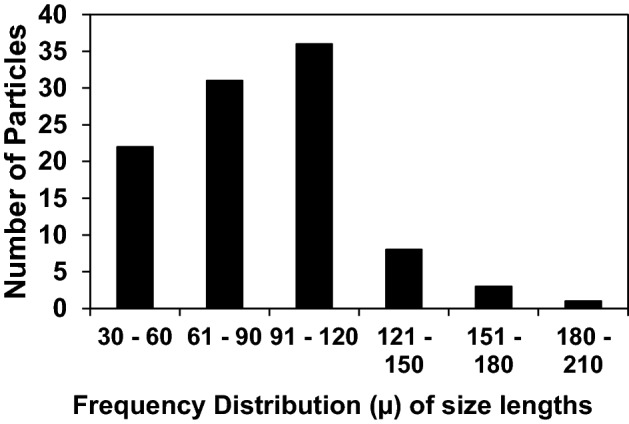
Frequency of observation of different size classes of polystyrene microplastics observed from 100 randomly selected particles over the study period.

Morphologically similar microplastics were first observed in water samples collected in March 2019. The polystyrene particles were transparent, bluish in color, curved in appearance, with uneven and straight fragmented edges (Fig. 2). FTIR compositional analysis of the particles showed that they were polystyrene (Fig. 3). The lengths of randomly selected polystyrene particles (n = 101) ranged from 33 to 190 µm, with a mean of 89.3 µm. The frequency distribution of lengths indicates that most polystyrene particles (n-101) had a length between 30 and 120 µm (Fig. 4). The size range of the polystyrene particles places them in a size range similar to microplanktonic algae, as defined by Sieburth et al.^16^, i.e. 20–200 µm. This is illustrated by the presence of the bioluminescent dinoflagellate *Pyrodinium bahamense*^28^ in Fig. 2a, next to the polystyrene particle. *P. bahamense* is a common feature of microplankton communities in the Florida Keys and other ecosystems in Florida^29,30^. Another image captured at lower magnification shows multiple polystyrene particles in the presence of another microplanktonic dinoflagellate species *Tripos hircus* (Fig. 2b).

**Figure 5:**
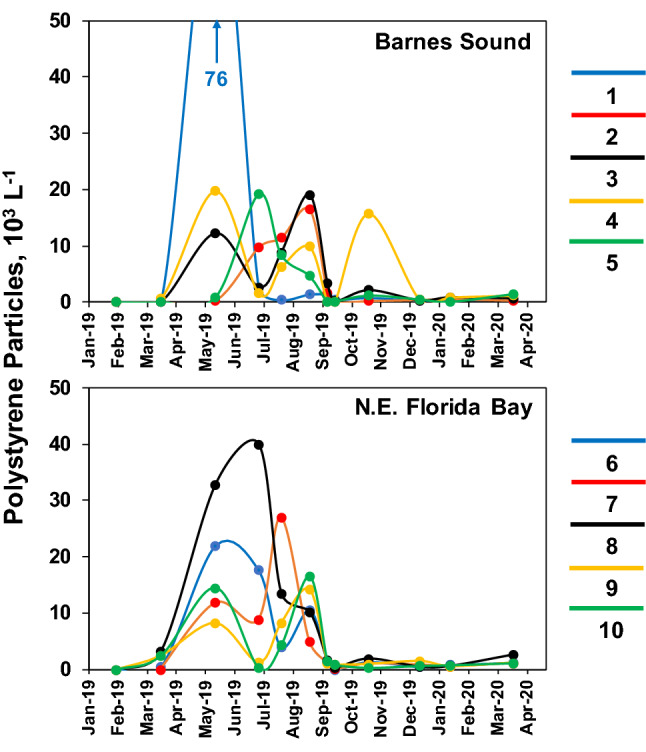
Concentrations of polystyrene microplastic particles at five sampling sites in Barnes Sound (1–5) and five sites in northeast Florida Bay (6–10) from January 2019 to March 2020.

Polystyrene microplastic particles were not observed during the first nine of twenty sampling events during the 2-year study period, i.e. May 31, June 27, July 26, August 16, September 6, September 19, October 4, and November 16 in 2018, and January 30 in 2019. The polystyrene particles were first observed on March 18, 2019 at six of the ten sampling sites, i.e. Sites 3 and 4 in Barnes Sound, and Sites 6, 8, 9, and 10 in northeast Florida Bay. Particle densities in the March samples ranged from 40 to 3,180 particles L^−1^, with the highest value at the eastern-most site (Site 8) in northeast Florida Bay (Fig. 5). In the May 15, 2019 sampling event polystyrene particles were observed at all ten sites and densities increased, reaching 76,000 particles L^−1^ at Site 1 in Manatee Bay (i.e. in northwest Barnes Sound) and 32,820 particles L^−1^ at Site 8 in northeast Florida Bay. During the following 3 months (June–August), polystyrene particles reached densities above 10,000 particles L^−1^ at all ten sites in at least one of the sampling events (Fig. 5). After August, particle densities were below 4000 particles L^−1^ at nine of ten sites, except for one high value at Site 4 in October.

**Figure 6:**
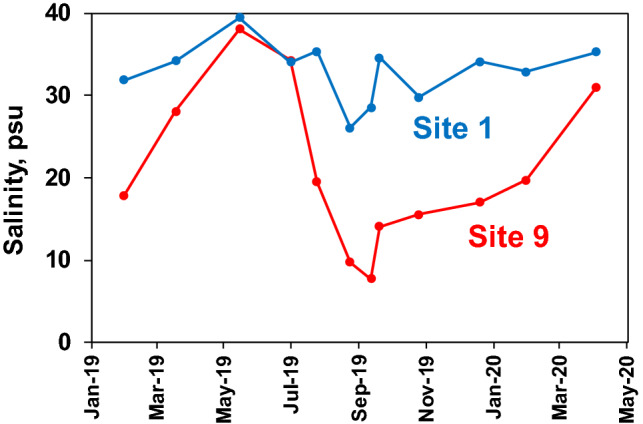
Salinities at Site 1 in Barnes Sound and Site 9 in N.E. Florida Bay from January 2019 through March 2020.

**Figure 7:**
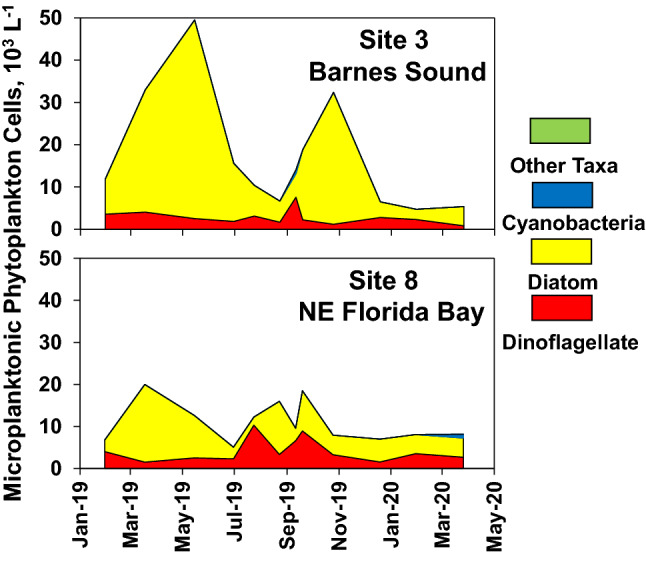
Examples of composition of microplanktonic phytoplankton (i.e. 20–200 µm) in Barnes Sound and NE Florida Bay in terms of cell density over the period when polystyrene microplastic particles were observed, i.e. January 2019 to March 2020.

Peak polystyrene particle densities were observed between May 2019 and August 2019 at all ten sites, and fell into a similar range of average cell densities as microplanktonic algae (i.e. cell size from 20 to 200 µm) during the same time period (Table 1). Mean polystyrene particle densities for the May–August period ranged from 8000 to 24,000 L^−1^ and mean microplanktonic algae cell densities ranged from 10,000 to 43,000 L^−1^ for the same time period. The period of peak microplastic polystyrene and microplanktonic algae densities coincided with the wet season in south Florida (i.e. May–October), during which salinities declined in both study regions (Fig. 6), due to rainfall and freshwater runoff from coastal watersheds and canals (e.g. C-111). Microplanktonic algae in the study regions include a diverse array of species, often numerically dominated by two functionally important groups of phytoplankton groups, i.e. diatoms and dinoflagellates^31^ (Fig. 7).

**Table 1:** Mean values for densities of polystyrene microplastic particles and microplanktonic algae cells over the peak period for microplastic densities, i.e. May–August 2019.

Site	Mean microplastic #		Mean microplankton #	
	10^3^ Particles L^−1^	Range	10^3^ Cells L^−1^	Range
1	20 (37)	0.4–76	28 (10)	13–36
2	10 (6)	0.3–17	17 (8)	9–29
3	11 (7)	2.7–19	21 (19)	7–50
4	9 (8)	1.5–20	10 (6)	4–18
5	8 (7)	0.9–19	11 (4)	6–15
6	14 (8)	4–18	14 (3)	11–17
7	13 (10)	5–27	13 (7)	5–23
8	24 (15)	10–40	11 (5)	5–20
9	8 (5)	1.2–14	43 (26)	18–44
10	9 (8)	0.3–17	29 (11)	13–37

Range of values for the period are also shown. Standard deviations are shown in parentheses.

## Discussion

This is the first report of widespread distribution and prolonged presence of small (< 200 µ) polystyrene microplastic particles in coastal lagoons of the Florida Keys. Polystyrene is widely used in construction materials, packaging foam, food containers, clerical supplies, medical equipment, fishing gear and many other applications^32,33^. Polystyrene has specific density (1.06 g cm^−3^) comparable to seawater (⁓ 1.02 g cm^−3^), and along with more buoyant polyolefin polymers, such as polyethylene and polypropylene, are common features of contamination in water columns of marine ecosystems^34,35^. Carpenter et al.^13^ found widespread distribution of polystyrene spherules in coastal waters of southern New England. Similarly, Dai et al.^36^ observed microplastic polystyrene particles in Bohai Sea along the coast of China. The uneven shapes and variable dimensions of the polystyrene microplastic particles observed in this study suggest that they are the result of degradation and/or fragmentation of larger debris, often referred to as secondary microplastics. The presence of secondary plastics is related to a range of degradation processes, including physical fragmentation or abrasion, photooxidation, chemical disruption or even biological processes^3,37,38^.

High concentrations of polystyrene microplastic particles were observed at all sites in both regions of the study, i.e. Barnes Sound and northeastern Florida Bay. The two regions are physically separated by land barriers with one connection via a navigational channel used for small vessels. The sudden and widespread appearance of high peak densities of the polystyrene particles raise questions about potential point and nonpoint sources of the material, and the basis for the high concentrations. While there is currently little information on potential point sources of the polystyrene particles in the study regions, it is possible that such sources exist. For example, Site 1 in the Manatee Bay region of Barnes Sound is located near the mouth of the C-111 Canal which drains coastal wetlands adjacent to urban, industrial and agricultural areas. In May 2019, Site 1 had the highest single density of polystyrene particles observed during the study period (76,000 L^−1^). May is the beginning of the wet season in Florida, when freshwater influences from the land are enhanced. Effluent discharges from regional industries and other commercial activities in the northern Florida Keys or adjacent mainland could explain the sudden appearance of high concentrations of the polystyrene microplastics. Southward flow of water from Biscayne Bay is also a potential source of microplastic contamination. However, there is insufficient information to define how the polystyrene microplastics spread to both Barnes Sound and northeastern Florida Bay, where high densities of the particles were observed in the same month.

Alternatively, the widespread distribution of high densities of polystyrene microplastic particles in both study regions at about the same time suggests the possibility of non-point-sources. The observation that the particles appear to be a product of degradation processes indicate that they may be a result of fragmentation of larger plastic debris. One potential source may be atmospheric transport from regional sources and aerial deposition (e.g., rainfall) into the study lagoons. This process has been previously reported involving microplastics in the size range observed in this study in several other regions of the world^39–41^. The process can result in the appearance of microplastics in remote and otherwise pristine areas of the world through long-distance aerial transport^42^.

Another possible non-point-source of the polystyrene may be related to Hurricane Irma, which caused widespread devastation in the Florida Keys and southern tip of the Florida peninsula in the fall of 2017. High winds of the Category 4 hurricane, along with flooding rainfall and storm surge, transported materials from residential, urban, industrial and agricultural land areas into northeast Florida Bay and southern Biscayne Bay, including Barnes Sound^43–45^. It is well-known that catastrophic events, such as hurricanes and tsunamis, contribute to the transport and deposition of plastic into the marine environment^11,46^. It is possible that polystyrene materials flushed into the study regions were entrained in shorelines, which are characterized by seagrass and mangrove communities. Plastic debris is frequently encountered in mangroves, where it becomes trapped and is subject to fragmentation^47,48^. Similarly, it has been suggested the sea grass beds act as traps for smaller plastics in coastal environments^49^. Polystyrene material trapped in these coastal communities are subject to fragmentation, ultimately producing smaller secondary microplastic particles of a size easily re-suspended and distributed by hydrologic processes into the water column. The timescale for fragmentation is variable depending on environmental conditions^2,11,50^. It is possible that fragmented polystyrene microplastics contained along shorelines of the study region after Hurricane Irma were flushed into the sampling regions by spring–summer rainfall events in the 2019 wet season in south Florida. Declines in salinity levels in coastal waters of Barnes Sound and northeast Florida Bay during the wet season reflect the influences of freshwater runoff.

The peak microplastic densities observed in this study were high compared to peak levels reported for some other marine ecosystems, such as the comparatively open waters of Liaodong Bay in China^36^, where observed peak microplastic abundance was 23 particles L^−1^. The high densities of polystyrene microplastics observed in northeast Florida Bay and Barnes Sound are in part related to the morphological and hydrologic characteristics of the study regions. The very shallow water depths (< 2–3 m) characteristic of these lagoons can enhance the density of particles, in part by increasing the potential for resuspension of the polystyrene particles. Small microplastic particles have low rise velocities and are susceptible to vertical transport^51^. Microplastics can spend extended periods of time in the water column and can be transported over long distances^15^. The abundance of small microplastic particles can also increase with decreasing depth and volume of the water column^2,52^. Analogous processes take place in microplankton communities. For example, diatoms, many of which fall into a similar size range and specific density as the polystyrene microplastics, rely on vertical mixing energy to maintain position in the water column^53^. Microplanktonic algae are important features of phytoplankton communities in Florida Bay^24,54,55^ and many other shallow estuaries in Florida^56–58^ and around the world^53^. Another feature contributing to the high densities of polystyrene particles is the restricted tidal flushing rates in the study regions. Shallow mudbanks and the land barriers represented by the islands that make up the Florida Keys limit water exchange between Barnes Sound and northeast Florida Bay and the Atlantic Ocean and Gulf of Mexico. Long water residence times in these regions can enhance the potential for accumulation and persistence of the polystyrene particles. Similar observations have been made for microplastics in other ecosystems^59^. Analogous observations have been made for microplanktonic algae abundances in coastal marine ecosystems with long residence times^25,30,60,61^.

The ubiquitous presence of polystyrene microplastic particles in Barnes Sound and northeast Florida Bay is a cause for concern because of potential impacts on aquatic organisms in the Florida Keys. Lusher et al.^18^ identified over 200 marine species, spanning a wide range of trophic levels, that ingested microplastics. The consumption of microplastic polystyrene spheres by zooplankton, important consumers of phytoplankton, has been shown to negatively impact their function and health, including altered feeding capacity, and a decrease in fecundity and survival rates^17,62,63^. In another study of zooplankton, including mysid shrimp, copepods, cladocerans, rotifers, polychaete larvae and ciliates from the Baltic Sea, there was transfer of microplastic particles from mesozooplankton ingesting polystyrene spheres to macrozooplankton^64^. The potential for bioaccumulation of microplastics in commercially important filter-feeding bivalves, such as oysters and clams, and other marine food chain components, represents a potential for human exposures to microplastics^12^. The small polystyrene microplastic particles (i.e. 33–190 µm) observed in our study fall into the same size range and abundance as microplanktonic algae (20–200 µm), highlighting the potential for consumption of polystyrene particles by the zooplankton community and other larger marine grazers, such as bivalves and sponges. An assortment of larger marine organisms, including fish, bivalves, and sea birds, have also been observed ingesting microplastics^65–67^. The ingestion of microplastic debris can introduce toxic substances to the food chain^19,68^. In addition, the sharp rigid edges of microplastic particles may cause physical damage to external structures of marine organisms, such as gill tissues of fish. The latter threat is similar to the potential physical damage associated with the presence of high densities of certain diatom species with rigid spines^69^.

Currently, the global extent of small microplastic particles of the size range observed in this study is likely under-reported, in part because some of the most common ways used to collect plastics, such as net tows, are not well adapted for capturing particles of that size range. Quantitative comparisons between the polystyrene microplastic particles and microplanktonic algae in this study reveal that abundances can reach similar levels that may potentially impact ecosystem structure and function. The high abundances and spatial distribution of small polystyrene microplastic particles observed in the Florida Keys highlight the potential for contamination of shallow restricted coastal ecosystems in ecosystems often considered to be relatively pristine, which may impact food web interactions^17,18,63,65,70,71^ and other microbial processes^20^. The potential breadth of impacts of microplastics certainly warrants further attention.

## Data Availability

The data used in this paper are part of project data reported to the South Florida Water Management District (W. Palm Beach, Florida) and should be accessible from the corresponding author, Edward Phlips, or the South Florida Water Management District (W. Palm Beach, Florida).
